# Semi-automated NMR Pipeline for Environmental Exposures: New Insights on the Metabolomics of Smokers versus Non-smokers

**Published:** 2021

**Authors:** Morris A. Aguilar, John McGuigan, Molly A. Hall

**Affiliations:** Huck Institutes of the Life Sciences, The Pennsylvania State University, 512 Wartik, University Park, PA 16802, USA; 512A Wartik Laboratory, University Park, PA 16801, USA

**Keywords:** Environmental Exposure, Metabolomics, Cigarette Smoke, Bioinformatics

## Abstract

Environmental exposure pathophysiology related to smoking can yield metabolic changes that are difficult to describe in a biologically informative fashion with manual proprietary software. Nuclear magnetic resonance (NMR) spectroscopy detects compounds found in biofluids yielding a metabolic snapshot. We applied our semi-automated NMR pipeline for a secondary analysis of a smoking study (MTBLS374 from the MetaboLights repository) (n = 112). This involved quality control (in the form of data preprocessing), automated metabolite quantification, and analysis. With our approach we putatively identified 79 metabolites that were previously unreported in the dataset. Quantified metabolites were used for metabolic pathway enrichment analysis that replicated 1 enriched pathway with the original study as well as 3 previously unreported pathways. Our pipeline generated a new random forest (RF) classifier between smoking classes that revealed several combinations of compounds. This study broadens our metabolomic understanding of smoking exposure by 1) notably increasing the number of quantified metabolites with our analytic pipeline, 2) suggesting smoking exposure may lead to heterogenous metabolic responses according to random forest modeling, and 3) modeling how newly quantified individual metabolites can determine smoking status. Our approach can be applied to other NMR studies to characterize environmental risk factors, allowing for the discovery of new biomarkers of disease and exposure status.

## 1. Introduction

Cigarette smoke (CS) is made of harmful constituents that cause many diseases.^1^ Additionally, there are many indicators that CS exposure has led to increased medical costs and loss of productivity over a lifespan.^2^ The thousands of reactive oxidative species (ROS) generated from burning cigarettes are found in the gaseous state and are responsible for CS related pathogenesis.^3^ The ROS damage epithelial cell linings by disrupting oxidative-sensitive metabolism and triggering DNA damage.^4^ The effects of CS on immunity can be both pro-inflammatory and suppressive.^5^ CS derived ROS can lead to neuronal damage^6^, atherosclerosis, increases predisposition of cardiovascular events^7^ and inhibit tumor suppressive mechanisms.^1^ Metabolomic interrogations of CS exposure may help investigators further understand the pathogenesis of several diseases strongly associated with CS exposure. Metabolomics studies the small molecules from biological samples that can reveal metabolic changes following environmental exposures.^8,9^ With respect to the genome, transcriptome, and proteome, metabolomics generally involves the small molecule compounds that are metabolized by enzymes; the metabolome can act synergistically with other “-omic” layers as well.^10^ Unlike other “-omics,” metabolomics reveals biochemical states and best represents the molecular phenotype.^8^ Additionally, metabolomic studies of disease can reveal new biomarkers, understudied pathways, and prognosis measures to improve risk stratification.^11,12^

A previous metabolomic study of CS that incorporated NMR and MS data derived from human blood serum found metabolites associated with chronic obstructive pulmonary disease, cardiovascular disease and cancer.^6,7,13^ This study by Kaluarachchi et al. is unique because it is the only study to date that used 1D 1H NMR on human blood serum for CS exposure from which 3 metabolites were reported.^13^ The raw NMR data for this human blood serum CS exposure study (n = 112) is publicly available on the MetaboLights repository as MTBLS374.^8,13^ The raw MTBLS374 data was originally analyzed with proprietary software to identify and quantify metabolites.

Although commercial software are popular, they often lack advanced editing, require iterative steps, and involve arbitrary adjustments based on subjective user judgement.^14^ Previous studies indicate that this manual method is prone to false positive metabolite identification that increases as more metabolites are quantified.^15,16^ The NMR analysis described here incorporates several R and Python packages to aid in the detection of additional metabolites that were previously unreported. We created novel random forest classification (RF) models from the quantitative metabolite data and the unprofiled spectra to classify smoking status. Furthermore, our RF classification decision trees reveal the statistical importance of the detected biomarkers, and findings were supported by pathway enrichment analysis.

Here we demonstrate how an environmental exposure like smoking and its metabolic effects can be quantified and modeled with NMR data via open source packages. With our pipeline, we quantified 79 previously undetected metabolites in this dataset. With the metabolite quantification data generated from our pipeline, we developed 2 high fidelity models that classified between the smoking classes. Our pipeline increases transparency of user set analysis parameters and unifies existing open source packages for spectral processing and multivariate analyses.

## 2. Methods

### 2.1. Data Set

The MTBLS374 dataset that was used for this study was acquired from the MetaboLights repository and contains 1D 1H NMR spectra of human blood serum from 112 participants.^8,13^ The original study also incorporated mass spectroscopy and lipoprotein fraction data in addition to NMR data to identify biochemical differences in smoking classes.^13^ They found that the metabolites they detected indicated that smoking exposure impacted glutathione, bilirubin and lipids. The authors suggested that their metabolic enrichment pathways were related to chronic obstructive pulmonary disease, cardiovascular diseases, and cancer.^13^ There were 55 (27 females, 28 males) smoker class samples and 57 (28 female, 29 male) never smoker class samples. The participants were from Hamburg, Germany who had a body mass index (BMI) within a healthy range and no clinical history of heart, lung diseases and chronic diseases. The MTBLS374 data set sample labels were limited to gender and smoking status (smoker/never smoker) due to adherence of participant privacy policies; however, the original study included BMI, age, and drug intake in their confounding analysis. The 1H 1D NMR spectroscopy data was generated with the Carr-Purcell-Meiboom-Gill pulse sequence with the following parameters: relaxation delay of 4 s, a mixing time of 0.01 s, a spin–echo delay of 0.3 ms, 128 loops and a free induction 3.067 s of decay acquisition time, total of 32 scans recorded into 96 thousand data points with a spectral width of 20 ppm.^13^

### 2.2. Pipeline

**Fig 1.**
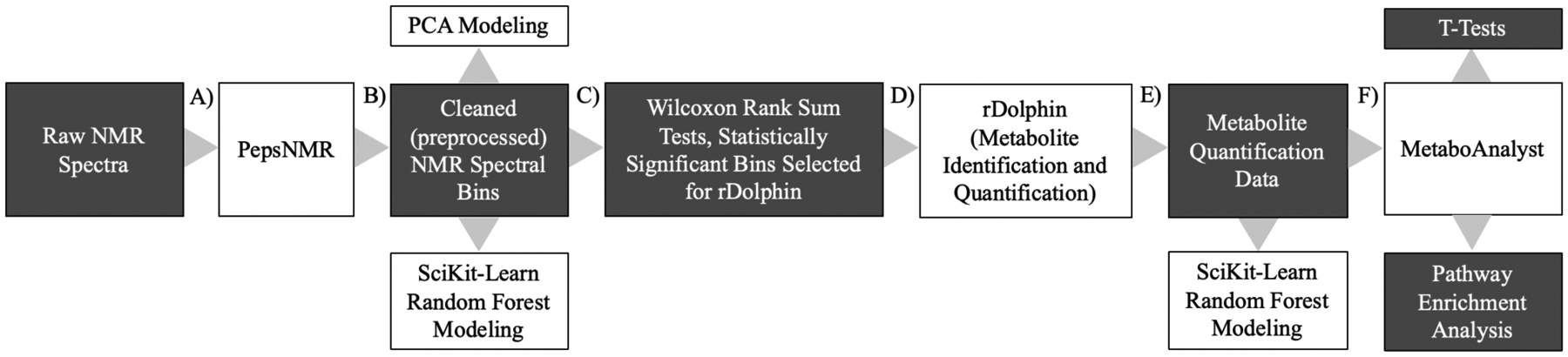
Semi-automatied pipeline for NMR based environmental exposure studies. The pipeline connected open source packages (white boxes). Outputs are represented in gray boxes.

The innovation of the pipeline lies in its capability of extracting metabolomic data from raw data NMR data in a semi-automated fashion (i.e., the arduous task of metabolite identification/quantification has been made automated, yet some parameter choices are still needed by the user). Open-source packages are unified to promote analysis reproducibility for the complex multistep analytical process of quantifying metabolic effects of environmental exposures. Typically, proprietary graphical user interface (GUI) software requires one set of software to edit the raw data to remove instrumental artifacts, a separate GUI application for metabolite quantification, and a separate statistical analysis software. These software do not record the repetitive and arbitrary user decisions to manipulate the data which is not conducive to analysis reproducibly. The proprietary software offers limited automation tools thereby constraining the user to iterative processes. The pipeline we describe here addresses the multiple steps data processing (Figure 1) and analysis challenges in environmental exposure metabolomics. We uploaded scripts to this pipeline to GitHub (github.com/HallLab/MTBLS374_smoking_study_secondary_analysis). We will describe the application of our pipeline to cigarette smoke exposure below.

#### 2.2.1 Preprocessing and Spectral Analysis

Before metabolites are identified and quantified, the first step in our pipeline is to preprocess the NMR data (i.e., data editing to enhance signal-to-noise ratio and minimize instrumental artifacts). This preprocessing is accomplished with the PepsNMR^14^ R package. A user may set parameters before bulk preprocessing of NMR data. The raw NMR data was first pre-processed (Figure 1a) so that the NMR data can be interpreted by subsequent analysis packages. The NMR spectra were zero-filled, Fourier transformed, zero phase corrected, first phase corrected, warping, binning, and normalized semi-autonomously by using the PepsNMR presets.^14^ We corrected for pH-induced chemical shifts with the warping and binning functions provided by PepsNMR. The NMR spectra were normalized with constant sum normalization which is recommended for sera.^14^ The regions corresponding to the water peak at 4.5 – 5.1 ppm were removed. The resulting output was preprocessed NMR data (Figure 1b) that can be utilized as input for subsequent analyses. Data clustering was observed with multivariate principal components analysis (PCA) analysis including samples who were categorized as smoking classes, and quality control class. The pre-processed binned spectral data was also used to generate random forest classification models with k-fold (k=10) validation with the Scikit-learn (0.22.1) python package.^17^

#### 2.2.2 Identification and Quantification

The binned spectral data were tested for significant spectral differences between the smoking classes. Between classes, each corresponding bin had a non-normal distribution thus warranting the Wilcoxon Rank Sum Test and Bonferroni adjustment (α =0.05) (Figure 1c).^18^ The spectral positions of the significant bins (Figure 1c) were cross referenced from a pure metabolite standard from HMDB to build a list of compounds that rDolphin^19^ (a profiling tool for 1H-NMR-based studies) automatically detects and quantifies within the preprocessed NMR data according to metabolite multiplicity and chemical shifts (Figure 1d).

#### 2.2.3 Analysis

The metabolite identification and quantification data output from rDolphin (Figure 1e) were used for t-tests and as features to train a second random forest classifier with k-fold (k=10) validation. The metabolite data was piped to the MetaboAnalyst R package (3.0.3) for data transformation such as normalization by sum, log transform and pareto scaling for t-tests (Figure 1f).^20^ Finally, the transformed metabolite data were used for metabolic pathway enrichment based on ontologies from the Kyoto Encyclopedia of Genes and Genomes (KEGG) Pathway Data Base and conducted via MetaboAnalyst. The enrichment analysis had 2-fold filter criteria.

## 3. Results

**Fig. 2.**
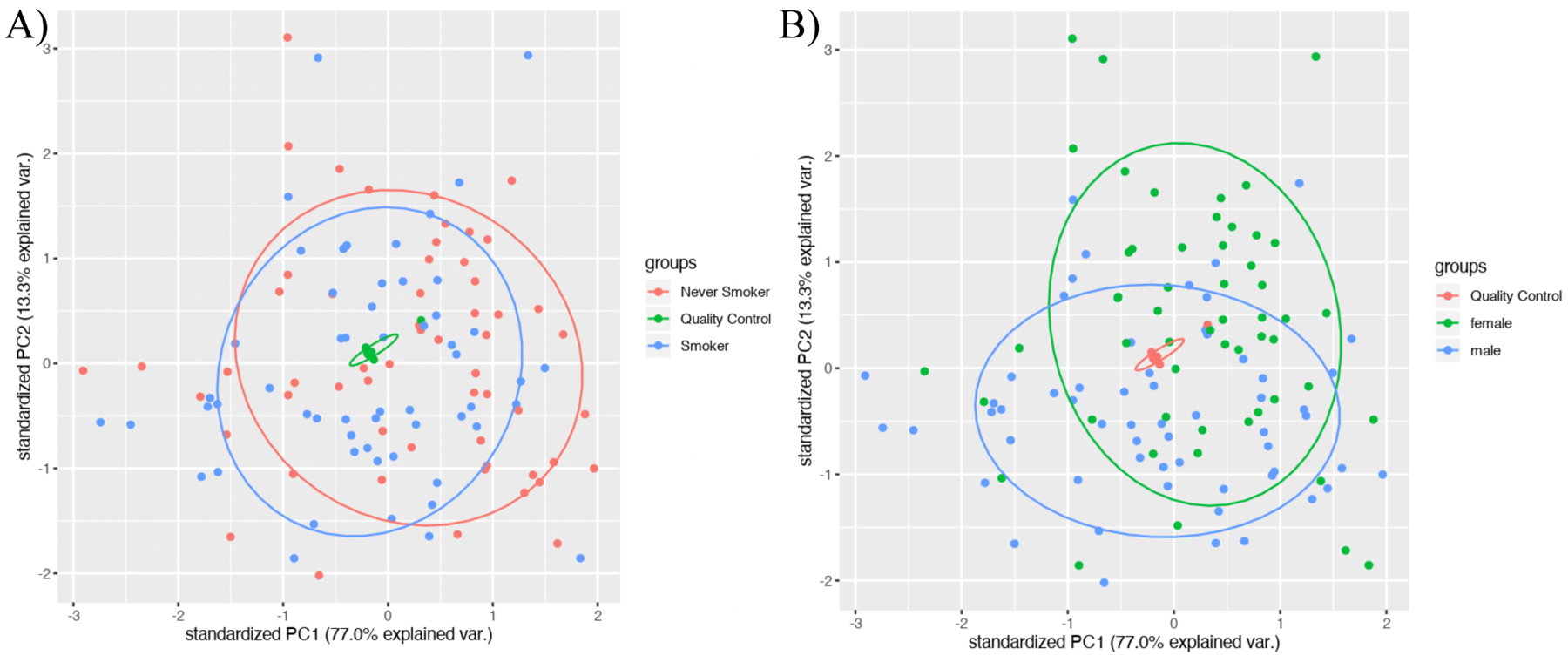
PCA Clustering of Smoking Status (A) and Gender Classes (B). PC 1 and PC2 are represented on the x-axis and y-axis, respectively. A) The PCA plot clustered the data points according to the female (green) and male (blue) classes according to PC 1 and PC 2. B) The PCA plot clusters the data points according to the smoker class (blue) and never smoker class (red). Both plots have a quality control class (red in subplot A and green in subplot B) to gauge technical variance.

A PCA was conducted on the pre-processed NMR spectral data to reveal clustering patterns based on smoking status and gender (Figure 2). Results from the PCA with the smoking classes indicate that the clusters overlap more so than the gender-based classes. PC1 and PC2 explain 77.0% and 13.3% of the variance for the gender and smoking status groups. The PCA results suggest that the gender classes may be a confounding factor. Logistic regression to test if gender was a significant predictor of smoking status in our data set yielded a non-significant (p-value: 0.40) predictor of smoking status.

**Fig. 3.**
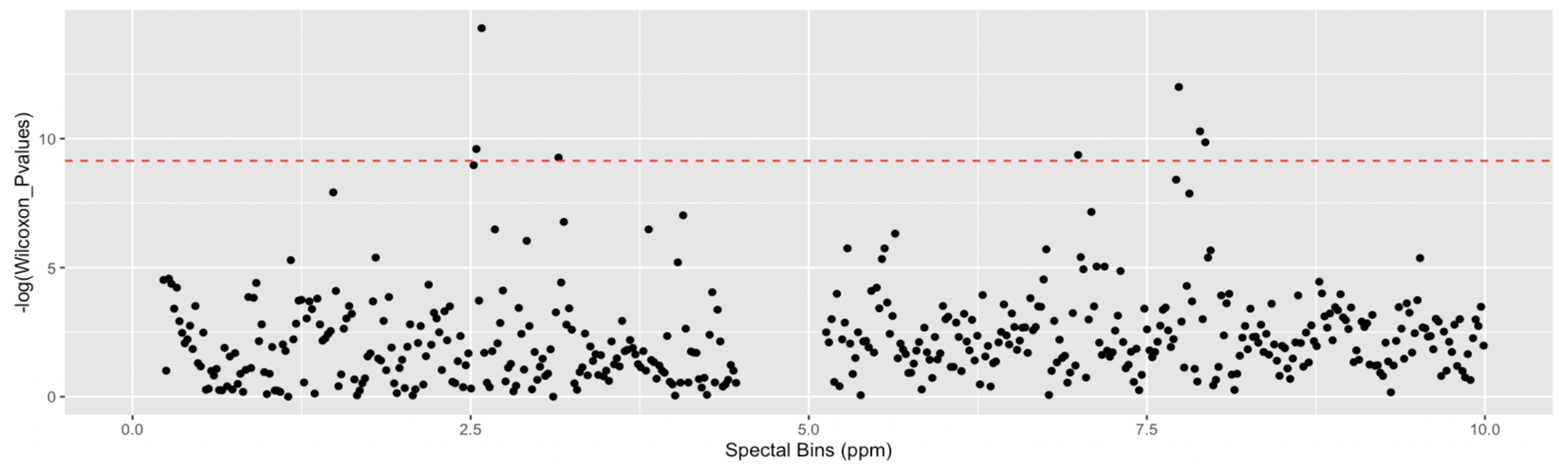
Manhattan plot of spectral bin associations with smoking status. The NMR spectrum for each sample was represented on the x-axis from 0 – 10 ppm and divided into bins with widths of 0.02 ppm and the y-axis represents the −log (10) of the p-value. The red line represents the Bonferroni significance threshold (alpha: 0.05, 467 tests). The absence of data points between at 4.5 – 5.1 ppm was expected due to the removal of the water signal.

To assess which NMR peaks warrant metabolite identification and quantification, the NMR spectral bins from 0.0 ppm to 10.0 ppm between the smoking classes were tested for significant differences in 467 spectral bins. For the Wilcoxon Rank Sum test, each bin was compared to its corresponding position in the NMR spectra between classes, i.e., the bin at position 1 ppm from the smoking class was only compared to the bin at position 1 ppm for the never smoker class. Each of the 467 non-normal spectral bins were tested for significance with the Wilcoxon Rank Sum test and 32 bins were significant when Bonferroni-adjusted (α: 0.05) (Figure 3). Spectral bins passing this threshold were investigated for metabolite identification and quantification via the rDolphin peak aligner.

**Fig. 4.**
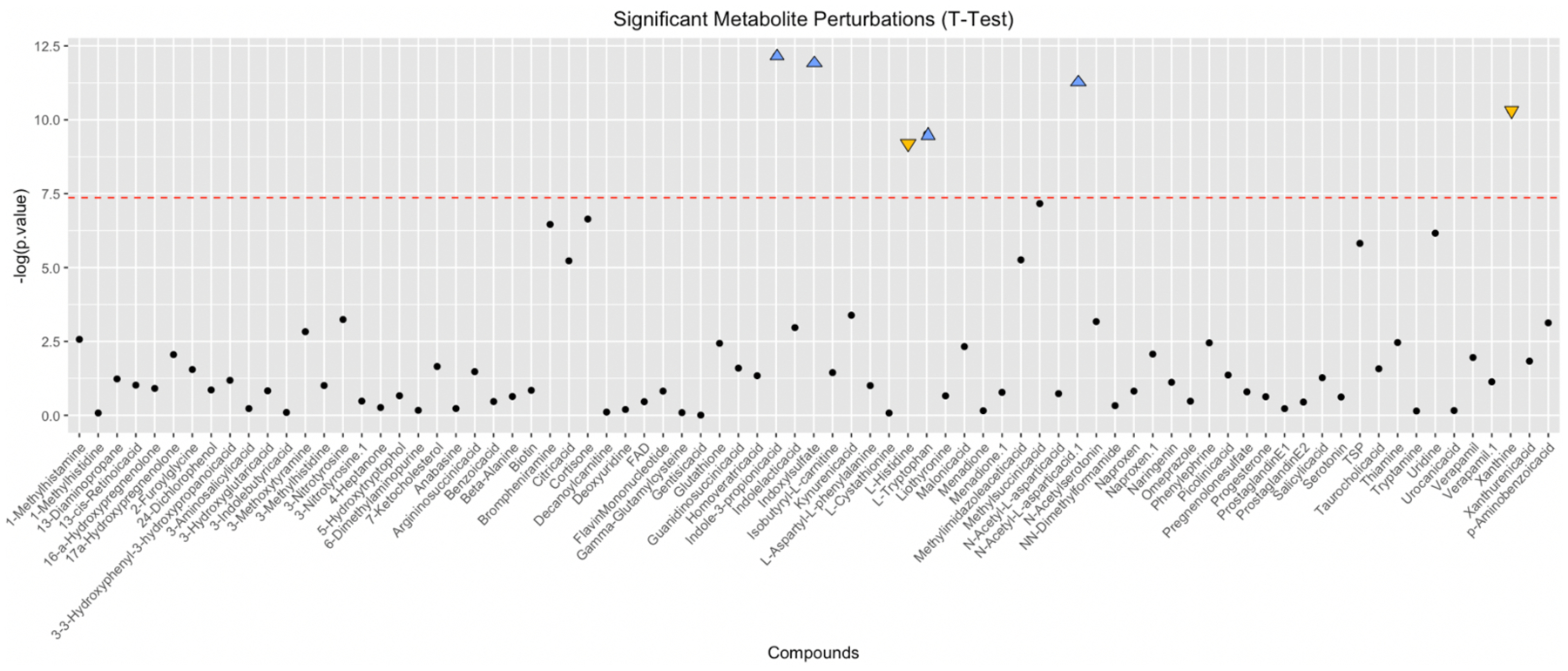
Manhattan plot of metabolite associations with smoking status. The Manhattan plot displays the metabolites on the x-axis and their −log(10) p-values on the y-axis. The red line represents the Bonferroni corrected significance threshold. The blue and yellow triangles represent increased and decreased metabolites.

After metabolite quantification, the 79 putatively identified metabolites and their relative concentrations were sum normalized, log transformed and pareto scaled for univariate two tailed t-tests. When the smoking classes were compared, 6 compounds were significant after Bonferroni adjustment (Figure 4). The significant metabolites include: Indole-3-propionicacid (p-value: 5.24 × 10^−6^), Indoxyl sulfate (p-value: 6.57 × 10^−6^), N-Acetyl-L-aspartic (p-value: 1.27 × 10^−5^), xanthine (p-value: 3.36 × 10^−5^), L-tryptophan (p-value: 7.36 × 10^−5^) and L-histidine (p-value: 0.00010336).

**Fig. 5.**
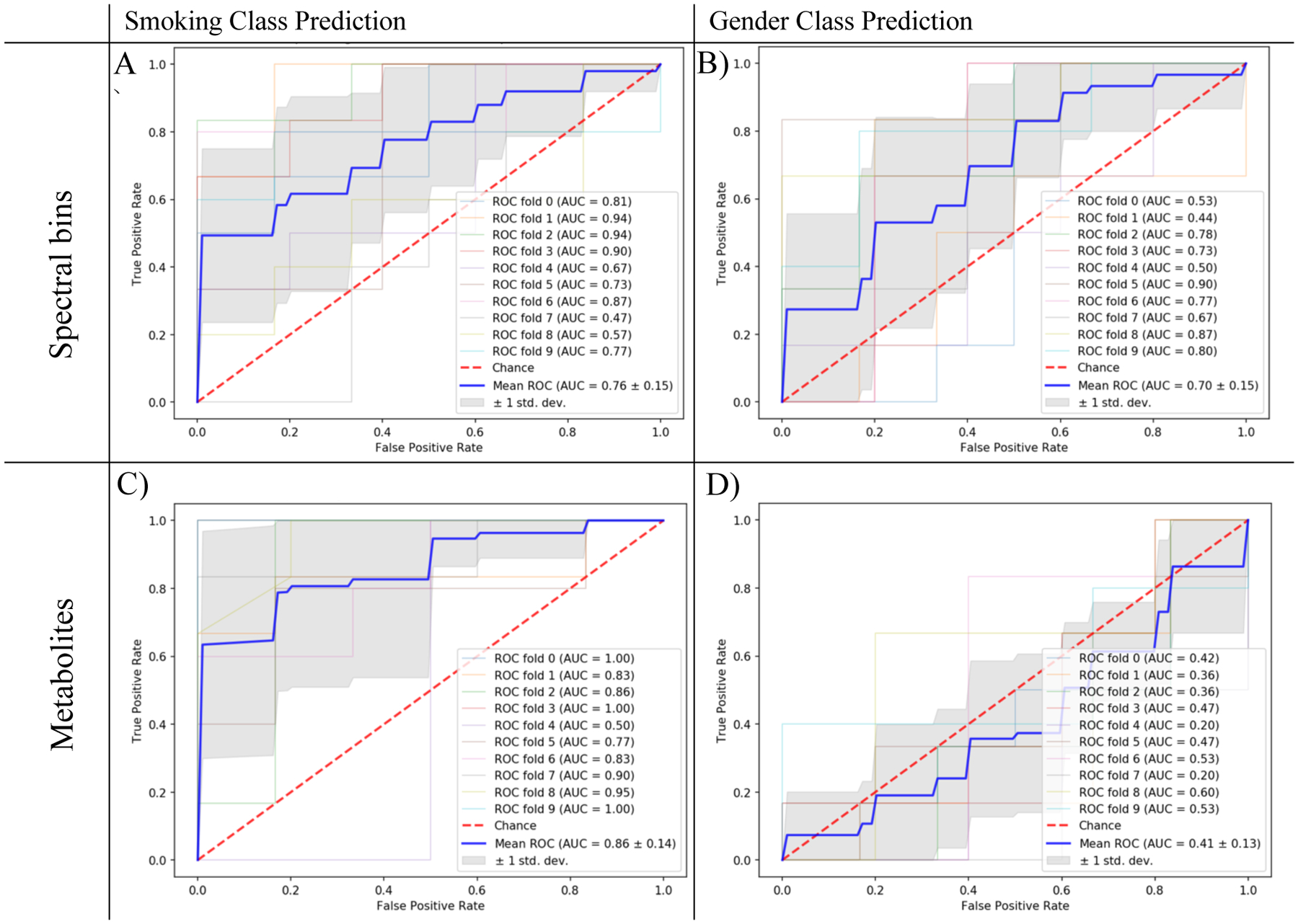
Smoking classes and gender classes prediction from spectral bins and metabolites. The ROC curves represent the RF models’ ability to discriminate between case and control and characterizes the model’s true positive and false positive rates. The plots also depict the model for every k-fold cross validation and the thick blue line represents the mean ROC curve derived from the cross validated models. A set of RF models were created by using the NMR spectral bins (467 per sample) as features. Another RF model set was created using the quantified metabolite data generated from the compound detection.

Two types of RF models were generated and were trained with either spectral data or quantitative metabolic data (Figure 5). For smoking status, the models demonstrated an AUC of 0.76 (SD: 0.15) for spectral bins (Figure 5a) and an AUC of 0.86 (SD: 0.14) for quantified metabolites (Figure 5c). For gender, the models demonstrated an AUC 0.70 (SD: 0.15) for spectral bins (Figure 5b) and AUC of 0.41 (SD: 0.13) for quantified metabolites (Figure 5d).

**Fig. 6.**
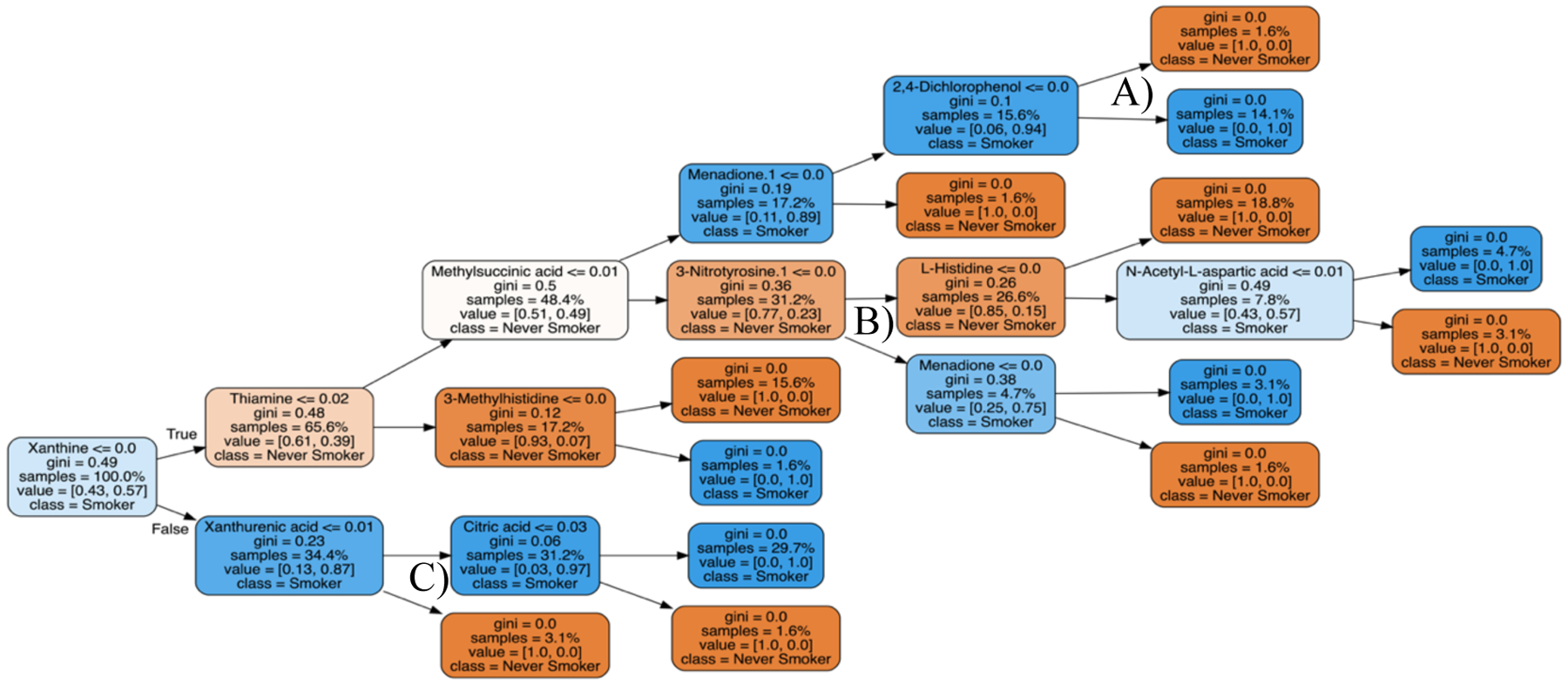
Metabolite random forest model for smoking classes prediction. This metabolite-based RF model has a decision tree that places each metabolite at a node and branches according to a Boolean quantitative threshold; when a condition was true the node branches upwards and if the condition was false the node branches downwards. Notable metabolites in the tree include A) 2,4-dichlorophenol, B) 3-nitrotyrosine, and c) xanthurenic acid. The decision tree emphasizes that several unique combinations of biomarkers differentiate smoking classes.

We created the decision tree from the RF model trained on the quantitative metabolic data that predicted smoking classes (Figure 6). When the RF model was trained it iteratively split the smoking classes into two branches but not all splits are perfect. Gini impurity represents the quality of the split between smoking classes at a node and a perfect split between classes at a node has a value of 0 like the terminal nodes in (Figure 6). 2,4-dichlorophenol (Figure 6a), 3-nitrotyrosine (Figure 6b), and xanthurenic acid (Figure 6c) have a gini impurity of 0.1, 0.36, and 0.23, respectively. The gini impurity at the 3-nitrotyrosine node indicates that the metabolite is not always perturbed for the smoking class which reveals smoking exposure metabolic heterogeneity. Also, at each node the percent of samples in the dataset that fulfill the quantitative threshold is given for each metabolite in the tree. The multivariate RF model indicates how combinations of metabolic perturbations occur depending on CS exposure which is more representative the highly interconnected metabolic biology of humans.

**Fig. 7.**
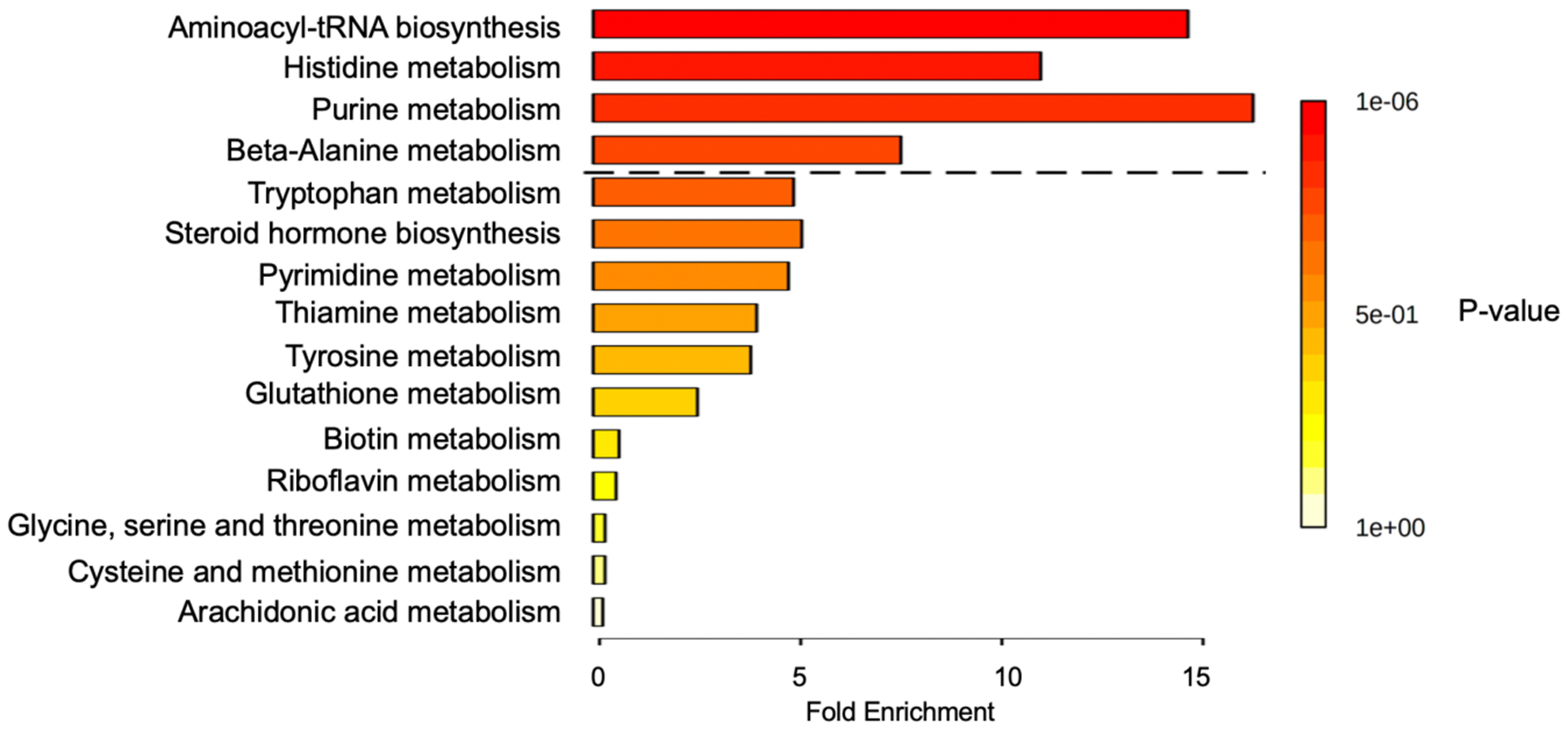
Metabolite enrichment overview. Metabolite enrichment analysis—with a 2-fold change criterion—from the KEGG Pathways data base reveals pathways that are enriched due to smoking status. The metabolic pathways above the black dashed line represents statistical significance after Bonferroni adjusted (α = 0.05) multiple test correction.

To determine which metabolic pathways were significantly perturbed, we performed enrichment tests on the 79 metabolites we quantified (that were found in the statistically significant spectral bins) and were mapped to known metabolic pathways from the KEGG database. The top 15 metabolic pathways that were perturbed between smoking classes are listed (Figure 7). The Bonferroni-adjusted significant pathways were aminoacyl-tRNA biosynthesis, histidine metabolism, purine metabolism, and beta-alanine metabolism. At most, the significantly enriched pathways have two metabolite hits which means that 2 of the metabolites we newly quantified are known to participate in that metabolic pathway.

## 4. Discussion

Environmental exposures can perturb the complex human metabolome, and it is difficult to quantify the numerous metabolic pathways with NMR data using proprietary software with limited automation features and no record of data transformation. We demonstrated the technical feasibly of describing the metabolome when affected by an environmental exposure like CS by unifying open source NMR packages. The MTBLS374 NMR data set was originally used to quantify 3 specific metabolites; however, the NMR spectra of each human blood serum sample was representative of thousands of metabolites that are expected to be found.^21^ We demonstrated our pipeline’s potential to increase the number of quantified metabolites.

To understand the global metabolomic differences between the smoking status classes and the gender classes, PCA was performed. The PCA cluster based on spectral data indicated more distinct separation between the gender-based classes than smoking exposure classes. The female and male groups have clusters that overlap with one another (Figure 1a), which suggests there may be more spectral differences related the metabolic sexual dimorphism which has been demonstrated previously.^22^ The pooled quality control classes clustered more tightly relative to the gender and smoking based classes, and we expected the quality control samples to display very little variance between one another and the variance that that we do detect likely came from variance from the NMR instrumentation.

We found 6 significant metabolites, all of which were not previously identified in the data set, however, we did only detect 1 out of the 3 metabolites the original authors found in the NMR data. We used a new computational approach involving semi-automated pre-processing and automated metabolite quantification open source packages as opposed to proprietary software like the original authors. Therefore, we did not necessarily expect to detect the same metabolites from the NMR data. Of the significantly perturbed metabolites from Figure 3, Indole-3-propionicacid is known to be neuroprotective antioxidant^23^ and more likely to be affected in smokers with atherosclerosis.^24^ Indoxyl sulfate is a known cardiotoxin and uremic toxin.^25^ A previous study found that indoxyl sulfate is lower in smokers’ blood serum, while here we found it was elevated.^26^ N-Acetyl-L-aspartic acid is one of the most concentrated compounds in the brain for myelin^27^ and a previous study found that this metabolite is decreased in the left hippocampus tissue in smokers.^28^ In our analysis we found that N-Acetyl-L-aspartic acid was elevated in blood serum. Xanthine is involved in the purine degradation pathway.^29^ The xanthine oxidase enzyme is elevated in smokers and it produces uric acid by consuming xanthine as a precursor molecule.^30^ We found that xanthine was significantly decreased in blood serum which might be due to its consumption of elevated xanthine oxidase. L-Tryptophan is an amino acid that is a precursor to hormones and neurotransmitters^31^ and has been found to be downregulated in those attempting to quit smoking.^32^ In our study we found that L-Tryptophan was significantly elevated which might play a role in cigarette smoking related behavior. L-Histidine is an essential amino acid and is a precursor to an inflammatory agent, histamine.^33^ L-Histidine is depressed in smokers without chronic obstructive pulmonary disease (COPD) versus those with COPD suggesting its consumption for histamine production thereby increasing inflammatory response.^34^ In our study, L-Histidine is significantly decreased suggesting that we might detect markers of inflammation in blood serum due to CS exposure. The significant perturbations of these 6 metabolites reinforces how CS exposure contributes to pathologies relating to ROS metabolism, cardiac damage, neural toxicity, and inflammatory response. Given that CS exposure perturbs individual metabolites it follows that it was possible to classify smoking exposure classes based on these perturbations.

The metabolite-based RF model that predicted smoking status has a decision tree that found novel relationships between metabolites. 2,4-Dichlorophenol (Figure 6a) is a known hazardous air pollutant and is a soil pollutant that tobacco plants can absorb.^35,36^ Within the context of other metabolites, 2,4-dichlorophenol is a necessary smoking class decision node. Smoking is associated with a decrease in 3-nitrotyrosine levels of plasma proteins and vascular endothelial dysfuction.^37^ 3-Nitrotyrosine (Figure 6b) was not significant within our univariate t-tests but in a multivariate context 3-nitrotyrosine was a necessary decision node for smoking classes. Although there is an inverse metabolic relationship between xanthine and neuronal uptake of xanthurenic acid^38^ on the path towards the terminal node (Figure 5c), there is no documented relation of these two metabolites with respect to smoking exposure. The root nodes in the decision tree (Figure 6) begin with a high gini impurity and terminate with 0 impurity. This means that each terminal node is dependent on the node path leading back to the root metabolite in the tree. In other words, these metabolite changes were dependent on one another to yield a metabolic profile indicative of the smoking classes. The combinations of these metabolites have not been previously documented and suggests a heterogenous response to a smoking exposure. These metabolite combinations used to classify smoking exposure status may be indicative of interconnected perturbations of metabolic pathways. Nevertheless, the decision tree found a statistical relationship and did not relate metabolites to mapped metabolic pathways.

We conducted a pathway enrichment analysis to relate how the metabolic perturbations we quantified relate to previously empirically derived metabolic pathways. In the enrichment analysis we included the 79 putatively identified compounds we quantified from NMR data. The original study found that the aminoacyl-tRNA biosynthesis was one of the top significantly enriched pathways which we replicated in this automated analysis. Another smoking exposure blood serum based mass spectroscopy study also corroborated the enrichment of aminoacyl-tRNA biosynthesis.^39^ Nonetheless we found purine, histidine, and biotin pathways to be enriched which was not previously described for human samples with CS exposure. These three pathways that we newly derived from NMR data is supported by a previous mass spectrometry blood serum based smoking study in a mouse model.^40^ A smoking exposure NMR study on mouse lung tissue extracts also found purine and histidine pathway perturbations likely due to cell injury.^41^ In particular the purine pathway perturbation might be due to CS related DNA damage and cell injury.^4^ The original study’s enrichment analysis was supplemented by mass spectroscopy data, which may contribute to divergence in enrichment results.

Although this study demonstrates that our pipeline can reveal more NMR generated metabolomic information about environmental exposures, we did not uncover all of the possible metabolic perturbations. The significant results from the univariate analysis described here provided a limited viewing window into the CS exposure metabolome because it does not describe the interconnected reality of human metabolism. The RF decision tree begins to describe interconnected metabolism and suggests that multiple combinations of metabolites are associated with the smoking classes. However, these combinations are not to be interpreted as being the only metabolites that are perturbed. Given that the public repository did not include the BMI, age, and drug intake data from the original study, we were not able to do additional confounder tests. Scalability of the pipeline becomes limited with data sets larger than MTBLS374 given that the preprocessing package (PepsNMR) and peak alignment package (rDolphin) where not coded with multicore support. Next steps include testing this pipeline on other NMR based environmental exposure studies to classify disease status, replicating major findings, and describing novel findings. Nonetheless, our unified pipeline overcame the limitations of manual NMR pre-processing and quantification and has enabled us to extract valuable metabolomic findings regarding smoking exposure.

## 5. Conclusion

Here we demonstrate how an environmental exposure like smoking and its metabolic effects can be quantified and modeled with NMR data. Our approach of filtering spectral bins via multiple tests informed which metabolites were automatically quantified. The RF modeling reveals how several unique combinations of metabolites are associated with smoking classes. This suggests there are more than one combination of metabolite perturbations associated with smoking and a heterogenous response to smoking exposure. Several of the metabolites that belong to these combinations have a known relationship to smoking and/or cellular damage. The novelty of our analysis approach lies in breaking from the conventional manual analysis methods and promoting study reproducibility.
